# Reducing Internet Gambling Harms Using Behavioral Science: A Stakeholder Framework

**DOI:** 10.3389/fpsyt.2020.598589

**Published:** 2020-12-14

**Authors:** Sally M. Gainsbury, Nicola Black, Alex Blaszczynski, Sascha Callaghan, Garner Clancey, Vladan Starcevic, Agnieszka Tymula

**Affiliations:** ^1^Gambling Treatment and Research Clinic, Brain and Mind Centre & School of Psychology, Faculty of Science, The University of Sydney, Sydney, NSW, Australia; ^2^Technology Addiction Team, The University of Sydney, Sydney, NSW, Australia; ^3^Sydney Law School, The University of Sydney, Sydney, NSW, Australia; ^4^Brain and Mind Centre & Sydney Law School, The University of Sydney, Sydney, NSW, Australia; ^5^Nepean Clinical School, Brain and Mind Centre & Faculty of Medicine and Health, The University of Sydney, Sydney, NSW, Australia; ^6^School of Economics, Brain and Mind Centre, Charles Perkins Centre & Faculty of Arts and Social Sciences, The University of Sydney, Sydney, NSW, Australia

**Keywords:** gambling (gaming), online, internet, technology, addictive behaviors, nudge design, behavioral science, persuasive design

## Abstract

Internet gambling provides a unique environment with design mechanics and data-driven opportunities that can impact gambling-related harms. Some elements of Internet gambling including isolation, lack of interruption, and constant, easy access have been argued to pose specific risks. However, identifiable player accounts enable identification of behavioral risk markers and personalized private interfaces to push customized messages and interventions. The structural design of the Internet gambling environment (website or app) can have a strong influence on individual behavior. However, unlike land-based venues, Internet gambling has few specific policies outlining acceptable and unacceptable design practices. Harm minimization including responsible gambling frameworks typically include roles and responsibilities for multiple stakeholders including individual users, industry operators, government regulators, and community organizations. This paper presents a framework for how behavioral science principles can inform appropriate stakeholder actions to minimize Internet gambling-related harms. A customer journey through internet gambling demonstrates how a multidisciplinary nexus of collaborative effort may facilitate a reduction in harms associated with Internet gambling for consumers at all stages of risk. Collaborative efforts between stakeholders could result in the implementation of appropriate design strategies to assist individuals to make decisions and engage in healthy, sustainable behaviors.

## Introduction

Gambling is a relatively common activity, however, for a minority of people gambling can lead to the development of gambling disorder, a mental disorder categorized as a behavioral addiction. Gambling disorder is highly co-morbid with other mental disorders and is characterized by a preoccupation with gambling and persistence and lack of control despite wide-spread negative consequences (1). Gambling problems may include sub-clinical but serious harms, which are experienced by 0.4–2.0% of adults internationally (2). Of those who experience gambling problems, the minority (7–29%) will seek treatment for these problems (3). The global online gambling market is expected to grow 13.2% between 2019 and 2020, from USD$58.9 billion to USD$66.7 billion (4). This growth appears to be due to COVID-19, which is limiting access to land-based gambling opportunities and resulting in more people gambling online.

Internet gambling occurs in a unique environment containing design mechanics and data-driven opportunities, with the potential to impact gambling-related harms. Just as the layout of land-based venues has been shown to influence gambling behavior (5–7), the design of websites has been shown to influence general ecommerce behavior (8). However, there has been minimal research investigating the impact of the design of Internet gambling websites. Some elements of Internet gambling, including isolation, lack of interruption, and constant, easy access, have been argued to pose specific risks (9). There is minimal research to guide evidence-based policies to design a sustainable online gambling environment in which individuals gamble at a level that is affordable for them and free from coercion or undue influence. We present here a framework for the role each key stakeholder can play in reducing harms from Internet gambling.

Persuasive design combines the theory of behavioral design with computer technology (10) and has been popularized by nudge theory (11). Nudge theory uses choice architecture and choice framing to ask questions in a way that nudges individuals' behavior in certain directions without restricting the available options–such as through opt-out default retirement funds. Systems of rewards and punishments in online gambling products are designed to encourage continued use and attention, additional payments, or other behaviors that are not always beneficial to the user, or consistent with their own plans and values. Examples include push notifications of time-limited promotional offers or matched deposits with complicated terms and conditions and limited benefits for users; excessive friction creating difficulty in withdrawing deposited funds; targeted push messages promoting a betting or spending options matching the user's profile (“people like you bet on…,”); and encouraging continuous use by eliminating natural breaks in play or the ability to pause (e.g., infinity scrolling). Most of these features are effective as they exploit natural human weaknesses in exercising self-control (12). In the heat of the moment, people often make decisions that favor immediate pleasure over later costs, in a way that is not consistent with their initial plans. Online gambling providers exploit this universal feature of human behavior to encourage more time and money spent on gambling.

On a positive side, behavioral science can identify nudges that steer users toward healthier levels of engagement with online gambling (which, for some people, may not include any gambling). Technological nudges are adaptable across settings with varying political and societal preferences around autonomy and paternalism, as the strength of the nudges can be adjusted accordingly. Software has been developed to monitor gambling and user activity, identify risk indicators, and enable well-timed interventions, including personalized, normative feedback, and encouragement to moderate play through pre-commitment devices (13–15). Dynamic messages can create a break in play and encourage self-appraisal (16, 17). Electronic gaming machines have been developed with customisable alarm clocks and ring-fenced winnings to prevent re-gambling (18). Digital wallets can limit gambling expenditure and provide personal feedback on gambling spend (19). Design options may include “plain packaging” for gambling sites (minimizing color and graphics), increasing friction by requiring users to click through different pages to access different betting/game options, creating pauses to slow the betting speed, reducing defaults bets, and requiring users to confirm bets and manually entering the amount, using default automated withdrawals of winnings, and default opt-out of notifications and marketing.

Policies based on behavioral science principles have been shown to be effective in influencing consumer behavior, including where personal risks are possible (20), although these have only recently been considered for gambling policies (21–23). This paper aims to present a framework for how behavioral science principles can inform appropriate stakeholder actions to minimize Internet gambling-related harm, with a focus on how technology can impact harms.

## Framework

There is a web of interacting factors that influence gambling related harms—including individual cognitive and personality characteristics of gambling users; various enticements and subtle influences used by gambling providers; cultural and social factors; availability of alcohol; and of course, individual choice. Opinions differ on who among those involved in the gambling experience ought to be responsible for reducing those harms. However, all those involved can, if they wish to, implement measures to do so.

**Figure 1:**
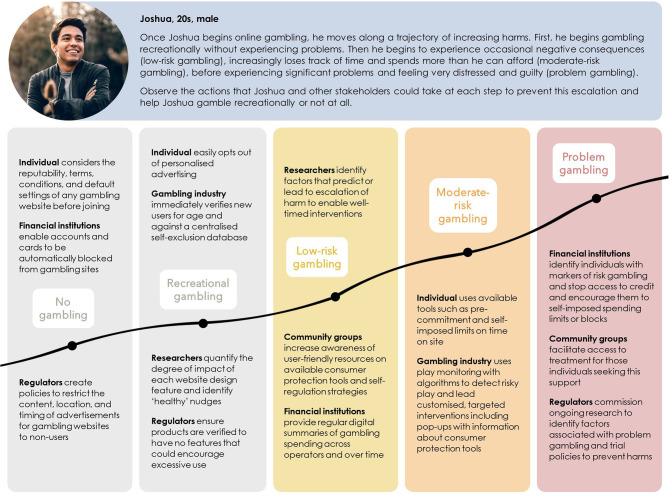
Hypothetical customer pathway illustrating appropriate stakeholder interventions according to level of gambling behavior and harm. For context, on average, 60–70% of individuals fall into some category of gambling behavior per year, with 1–2% falling in the “problem” category; however this varies between jurisdictions (25).

Customer journey maps visually represent user experiences in using services such as gambling websites (24). We use this method in Figure 1 to illustrate (1) a hypothetical journey of escalating harms from online gambling that a customer, “Joshua” could take, and (2) the roles different stakeholders could play at each step of the journey in order to alter its course toward a lower level of harm. We intend this map to highlight pivotal points from a user perspective and provide tangible calls to action for all stakeholders.

### Individual Users

There is a range of actions that individuals can take to decrease the chance that their online gambling behaviors cause harms for themselves, their families, and their communities. Individuals should inform themselves about the risks and persuasion associated with website features. With such knowledge, individuals will be better placed to select regulated websites that employ responsible design, to turn off any default persuasive design elements, and to select the settings they prefer. This could include disabling features that nudge users toward continued gambling. At the same time, some individuals will find it difficult to make informed decisions about gambling due to factors such as comorbid conditions, addiction, or impulsivity that make it more difficult to exercise self-control. This speaks to the necessity of this broader framework that identifies roles for multiple stakeholders.

Similarly, individuals should inform themselves about tools available to reduce harms. These include consumer protection tools such as self-exclusions and limits (26), but may include more general self-regulation tools that can be implemented in any behavioral domain to reduce the need to exercise self-control in the moment (27). Apps and software can be used to limit and restrict access to specific apps/websites, and limits can be placed on payments and access to credit. Users may avoid features that minimize friction to provide greater opportunities for self-reflection. For example, by avoiding options to remain signed-in to accounts for betting and avoiding saved passwords, requiring manual entry of passwords. At the beginning of a gambling session, an individual may set a timer on their device with an alarm to subsequently signal the planned end of the gambling session. Such strategies are only likely to be adopted by individuals who are motived to regulate or reduce their gambling (27, 28). Other individuals will likely view these strategies as a hindrance toward their goal of gambling, which might be meeting needs for relatedness, competency, or mood modulation (29–31). Knowledge of available tools combined with a desire or willingness to use them might be helpful to minimize the intention-behavior gap and self-control issues (27, 28). There are many tools available to assist individuals to enforce their planned behaviors if they have the knowledge and motivation to use these and autonomy to make informed choices.

### Community Groups

Community groups are typically non-profit organizations (may be large or small and focus on broad or specific issues or target groups) that are established and operated independently from governments and are typically funded from a range of stakeholders, commonly governments or charity donations. These groups have the capacity to provide education and outreach to communities, mobilize resources, advocate for citizens, challenge policy, and conduct various projects to impact communities. Community groups can collaborate with other stakeholders to reach shared goals, such as working with researchers to create and disseminate up-to-date communication materials about risks and protective strategies in formats that are accessible to individuals. In collaboration with researchers, community groups might also provide tools to individuals to help them understand their own personal risks of gambling harms, such as self-assessment quizzes with personalized feedback. These strategies might help to shift their individual attitudes toward gambling (28). Community groups can work in an advocacy role to convey the needs and concerns of individuals to regulators. Efforts are needed to ensure funding received from stakeholders is provided in an independent manner without restrictions and involvement by the funding body to minimize conflicts of interest and funding should not be reliant on gambling expenditure.

### Gambling Industry

The gambling industry is responsible for ensuring that websites, apps, products, offers, marketing, and communication are designed to facilitate the customer's need for autonomy (31), encourage gambling only at personally affordable levels, and reduce the risk of foreseeable harms. Operators should avoid using overly persuasive design elements as this violates the principles of autonomy and informed choice. Features to avoid could include those which create a sense of urgency (e.g., countdown timers on bets and promotions), that distort attitudes by creating overly optimistic perceptions of the chance of winning or reduce the perceived likelihood of losing (e.g., dynamic leader boards of recent winners, money back guarantee bets) (28), providing irrelevant information that perpetuate erroneous beliefs (e.g., providing details of previous wins in independent events such as winning lottery or roulette numbers, time since last jackpot, location winning lottery tickets were sold) (28), promoting irrelevant information to perpetuate social norms (e.g., most popular bets, number of active users) (28), or that act to reduce the opportunity to reflect on the decision to place a bet or make a deposit (e.g., prompted bet size, frictionless betting).

Gambling industry operators have a responsibility to “know their customer,” to verify a customer's identity prior to accepting any bets, and to avoid exacerbating any harms experienced by customers who are identified as at-risk or already experiencing gambling-related problems. Verified player accounts enable identification of behavioral risk markers and personalized private interfaces to push customized messages and interventions (32, 33). For example, operators could delay sending promotional offers until they have a good understanding of their customers and use continuous monitoring programs and algorithms to identify customers with risk indicators and respond appropriately with messages to encourage use of consumer protection tools, phone calls to check in with customers, or automatic blocking of promotions and marketing materials (26).

In addition to the avoidance of harm (principle of nonmaleficence), website operators also have the opportunity to do good for their customers (principle of beneficence) (34). The gambling industry could implement consumer protection tools as the (modifiable) default option. For example, a time “limit” could be placed on all users, whereby a message alerts users when they have gambled for the limited time, and requires users to change the default settings if they wish to gamble for longer. Users could be shown pop up displays summarizing their behavior in comparison to that of other users (personalized, normative feedback) thereby potentially shifting their attitudes and social norms (28), directing the user to information about consumer protection tools that are available to them (e.g., spending limits and self-exclusion), and creating friction by using pop-up messages and breaks in play to prompt the user to pause and reflect (e.g., please confirm that you want to place your xth bet for this week) (35, 36). To preserve autonomy (31), customers should be able to turn on (opt-in to) notifications and marketing and turn down (opt-out of) restrictions such as deposit limits; however, by making these active choices operators are prompting sustainable gambling–that is, gambling within their financial means and without associated harm/s. To ensure they are effective and well-received, the exact content and delivery of interventions should be negotiated in collaboration with other stakeholders–particularly users and researchers.

### Government and Regulators

Like industry operators, governments and regulators have a responsibility to ensure that all legalized products and activities contribute to the public good and do no harm. Governments should consider approving non-exploitative forms of gambling, as well as consumer protections. Regulators and policy makers have a responsibility to commission research to guide the development of policy options, review evidence to inform these, and seek consultation from other stakeholders and the public, to ensure that industry standards conform to social expectations. As technology continues to evolve, it is likely that commissioned research will be needed to analyse of the impacts of individual website features and assess those impacts for harm. Experience from venue-based gambling regulation could also be expected to inform online gambling regulation where the former includes regulation of ambient and other factors that create unacceptable risks for gambling users. Regulatory and policy direction is increasingly focusing on online gambling as it steadily increases as a proportion of gambling activity. As with all tech regulation, the challenge will be to create policies that are specific enough to be effective, but also future proof. As the gambling environment is impacted by multiple layers of regulation, across jurisdictions, inter-governmental coordination on the relevant issues will also be critically important.

### Financial Institutions

Financial institutions including banks and credit providers are able to contribute to reducing harms from online gambling by providing consumer tools to assist individuals to manage their online gambling spending and using algorithms to identify indicators of risky gambling (37). Financial institutions could provide individuals with comprehensive activity and expenditure statements collating all gambling spending in one place and as a proportion of income and discretionary expenditure. Statements could be an easily accessible way to communicate to customers evidence of risk indicators such as increased gambling spend or frequency in relation to previous time periods and relative to income and other expenses. Financial institutions could provide products with voluntary or default gambling spend limits or blocks and notify customers as they are approaching their limits. Non-gambling products could be developed and marketed to those who wish to opt-out of gambling completely, such as for adolescents and those who identify themselves as at-risk due to their personal situations. It is difficult for financial institutions to limit customers in spending their own money; however, there may be a duty of care implication related to offering credit to customers for the purposes of gambling given the demonstrated relationship between consumer debt and gambling problems (38, 39).

### Researchers

There is a role for researchers across academic disciplines in working together to ensure the evidence supporting each element involved in reducing harms from online gambling is robust. Research should focus both on the elements of the online environment (and their interactions with user characteristics) that can cause harm, as well as mechanisms of harnessing technology to prevent harm. Research should investigate mental health issues specifically associated with online gambling. These can contribute to functional impairment and include depression, suicidal behavior and proneness to psychoactive substance misuse, among other issues. Cross-disciplinary researchers can use behavioral economics theory and apply a variety of methods to identify the existing persuasive elements of the online gambling design, identifying nudges that will help maintain healthy levels of gambling without restricting autonomy of the players (31), as well as quantifying the degree of impact of persuasive design features on gambling behavior and harms. Reliable indicators of the size of effects of different features are needed to inform good policy about their use and to identify priority areas for policy development. Specific attention could be paid to those features already in use, such as financial incentives (40), time-sensitive promotions (41), targeted advertising, default site settings, and displays of “latest winners” (41).

Researchers can use the existing data to create models that will identify at-risk individuals from their usage patterns before life-changing harm occurs. In collaboration with industry operators and financial institutions, this research could inform algorithms to identify at-risk individuals in practice and deliver automatised, personalized intervention or prevention strategies. Research should focus on the multiple harms related to online gambling. The intersection between online gambling, fraud, theft, and violence-related offenses, for example, could usefully be explored by criminologists. Such insights will help in arguments regarding policy and regulatory responses required to minimize harms.

For maximal real-world impact, researchers across disciplines must be responsive to the needs and opinions of the other stakeholders with respect to priority research areas. This could involve proactive involvement of stakeholders into research design and dissemination and implementation of findings, as well as reactive design of research to address issues identified by other stakeholders. This will ensure that the research being conducted continues to address evolving real-world problems. All stakeholders should work with researchers to develop, test, and evaluate policies and strategies designed to minimize harms and to check for any unintended negative consequences.

## Conclusion

This paper aimed to describe a framework of opportunities by which different stakeholder groups can contribute to the shared goal of reducing harm associated with online gambling. The value of this framework is that it makes explicit the roles and responsibilities of each stakeholder. In addition to those roles listed above we propose open and transparent collaborative communication between stakeholder groups as a role for all stakeholders. This is particularly important in the field of (Internet) gambling when stakeholders can hold competing interests. For example, operators' commercial imperatives compete with their need for corporate social responsibility and duty of care. Taxation revenue benefits must be balanced against governments' need to minimize harm caused by legal activities. Users face a conflict between possible long-term harms and short-term enjoyments. Community groups need to balance the needs of a minority who experience significant gambling-related harms with those who enjoy gambling and want to make autonomous choices. We intend this framework to be a step toward acknowledging and mediating these competing interests. This framework is intended to be preliminary and to facilitate discussion. As such, we welcome comments on further roles not described here that any of these stakeholder groups could play as well as suggestions of other stakeholder groups who could play a role in reducing online gambling harms. We also hope that it will serve as a structured outline of the types of harm-reduction strategies that warrant further investigation to determine their effectiveness, as this empirical evidence is somewhat limited with respect to Internet gambling.

Practical steps can be taken to achieve collaboration between stakeholders to reduce Internet-gambling-related harms. Actions that facilitate communication between stakeholders could include conferences and roundtables dedicated to this purpose. Such events will increase the knowledge held by each stakeholder of the others' roles, values, and motivations, which will ultimately lead to more effective communication. Co-funding, co-design, and co-evaluation of projects are further ways in which stakeholders could make tangible strides toward the shared goal. Behavioral science principles respect individual autonomy, allowing modifiable restrictions to be used to protect the at-risk minority. They may be imposed by regulators or implemented by operators as a form of self-regulation and corporate social responsibility, or even a marketing strategy to attract customers. In any case, design strategies can assist individuals to make decisions and act in ways that contribute to a healthy and sustainable lifestyle and overall wellbeing.

## Data Availability Statement

The original contributions presented in the study are included in the article/supplementary material, further inquiries can be directed to the corresponding author.

## Author Contributions

SG and AB conceived of the paper idea. SG and NB drafted the manuscript. All authors contributed to and revised the manuscript critically and approved the final version for submission.

## Conflict of Interest

AB has conducted research funded directly ClubsNSW. He is also on the responsible gambling advisory panel for Crown Casino. Over the last three years (2017–2020), SG has worked on projects that have received funding and in-kind support through her institution from GameCo, ClubsNSW, Crown Resorts, and Wymac Gaming. SG has received honorarium and travel costs for research, presentations, and advisory services from Credit Suisse, ClubsNSW, Clubs4Fun, Clayton Utz, Greenslade, Generation Next, KPMG, Stibbe, QBE, Stiftelsen Nordiska Sällskapet för Upplysning om Spelberoende. The remaining authors declare that the research was conducted in the absence of any commercial or financial relationships that could be construed as a potential conflict of interest.
